# DCAF12 and HSPA1A May Serve as Potential Diagnostic Biomarkers for Myasthenia Gravis

**DOI:** 10.1155/2022/8587273

**Published:** 2022-05-24

**Authors:** Weidong Nong, Fang Huang, Fengping Mao, Dayuan Lao, Zhuowei Gong, Wen Huang

**Affiliations:** Department of Neurology, First Affiliated Hospital, Guangxi Medical University, Nanning, China 530021

## Abstract

**Background:**

Myasthenia gravis (MG) is an autoimmune disease that severely affects the life quality of patients. This study explores the differences in immune cell types between MG and healthy control and the role of immune-related genes in the diagnosis of MG.

**Methods:**

The GSE85452 dataset was downloaded from the Gene Expression Omnibus (GEO) database and analyzed using the limma package to determine differentially expressed genes (DEGs) between patients with MG and the control group. Differentially expressed immune cells were analyzed using single-sample gene set enrichment analysis (GSEA), while immune cell-associated modules were identified by weighted gene coexpression network analysis (WGCNA). Then, the expression of the identified hub genes was confirmed by RT-PCR in peripheral blood mononuclear cells (PBMCs) of MG patients. The R package pROC was used to plot the receiver operating characteristics (ROC) curves.

**Results:**

The modules related to CD56^bright^ natural killer cells were identified by GSEA and WGCNA. The proportion of CD56^bright^ natural killer cells in the peripheral blood of MG patients is low. The results of RT-PCR showed that the levels of DDB1- and CUL4-associated factor 12 (DCAF12) and heat shock protein family A member 1A (HSPA1A) were significantly decreased in peripheral blood mononuclear cells of MG patients compared with healthy controls. The ROC curve results of DCAF12 and HSPA1A mRNA in MG diagnosis were 0.780 and 0.830, respectively.

**Conclusions:**

CD56^bright^ NK cell is lower in MG patients and may affect MG occurrence. DCAF12 and HSPA1A are lowly expressed in PBMCs of MG patients and may serve as the diagnostic biomarkers of MG.

## 1. Introduction

Myasthenia gravis (MG), characterized by cellular immune participation and complement involvement, is a group of chronic autoimmune diseases mediated by antibodies targeting the neuromuscular junction (NMJ). MG results in disturbances in NMJ transmission, manifested by fluctuating fatigue-prone skeletal muscle weakness. MG decreases the patient's quality of life, with many cases requiring hospitalization. Severe cases can be life-threatening.

As an autoimmune disease, the imbalance of immune cells in the body is one of the most critical factors leading to the pathogenesis of MG. Various immune cells represented by T cells, B cells, dendritic cells, and natural killer cells participate in and mediate the autoimmune response of myasthenia gravis [1–4]. These imbalances of immune cells induce proinflammatory cytokines and autoreactive antibodies, which destroy the structure and function of the NMJ. Therefore, studying the pathogenesis of myasthenia gravis from the perspective of immune cell disruption is a prerequisite for the targeted clinical decision-making and treatment of patients with myasthenia gravis. It is the current hot spot and research trend for myasthenia gravis [5], which can be helpful in elucidating MG and provides new ideas and new approaches for clinical diagnosis and treatment.

The current understanding is that the presence of specific circulating autoantibodies targeting acetylcholine receptor (AChR), muscle-specific tyrosine kinase (MuSK), or low-density lipoprotein receptor-related protein 4 plays essential roles in the immunopathology of myasthenia gravis. Serological tests for these autoantibodies are the laboratory procedure most frequently used to confirm an MG diagnosis and classify subgroups of the disease. Although serological autoantibody testing was relatively easy and noninvasive for diagnosing MG, approximately 10% of patients with MG were seronegative with the classical commercial serological diagnostic tests [6]. In addition, their titers are not necessarily associated with disease severity or response to treatment. Despite increasing recognition of the pathogenesis of myasthenia gravis, the precise immunological mechanisms leading to the chronic development of persistent autoimmunity in patients with myasthenia gravis are not clear. Moreover, refractoriness to treatment is commonly seen in some MG patients. Early diagnosis of MG, along with a timely and effective treatment plan, will result in a better prognosis. MG can mimic various neurological disorders, leading to delays in diagnosis and treatment. Delayed diagnosis of MG is associated with increased mortality, especially in patients with late onset [7]. The differences in treatment response among MG patients highlight the need to identify new additional biomarkers to supplement the existing diagnostic toolkit.

## 2. Materials and Methods

### 2.1. Data Collection and Processing

The GSE85452 dataset of publicly available MGs was downloaded from the GEO database (http://www.ncbi.nlm.nih.gov/geo/) database using an R package GEOquery as the training set. It contains the mRNA expression profiles of 13 MG samples and 12 normal samples. According to previous research [8], the data of the thymoma sample were downloaded from TCGA database (http://tcga.cancer.gov/dataportal) as a validation set (TCGA-THYM), including 11 MG samples and 10 normal samples. The R limma package was used to identify differentially expressed genes (DEGs) between the expression profiles of MG and normal samples with the screening criteria: |logFC| > 0.5 and the *P* value < 0.05. The ggplot2 and pheatmap R packages were used to visualize the expression patterns of DEGs by volcano plot and heatmaps.

### 2.2. Single-Sample Gene Set Enrichment Analysis

The GSVA R package was used to quantify the enrichment fraction of 28 immune cell species per sample in the GSE85452 dataset. Based on the enrichment fraction matrix of the single sample gene set enrichment analysis (ssGSEA), immune infiltrating cells with significant differences in enrichment scores between the normal and MG groups were analyzed (*P* < 0.05).

### 2.3. Weighted Gene Coexpression Network Analysis

A gene coexpression network was constructed by weighted gene coexpression network analysis (WGCNA) based on the gene expression profile data of samples in the GSE85452 dataset. Then, the gene modules were divided by hierarchical clustering. Pearson's correlation analysis was used to calculate the correlation between gene modules and immune cells. The module with the highest correlation was selected as the key gene module. After identifying the key modules, each gene's gene significance (GS) and module membership (MM) were calculated. The key genes within the modules were selected according to the thresholds of GS > 0.8 and MM > 0.8. These genes were named immune-related hub genes.

### 2.4. Functional Enrichment Analysis

ClusterProfiler was used for the GO and KEGG enrichment analysis. GO described gene function in three aspects: biological process, cell component, and molecular function. The thresholds for the GO enrichment entries were adj. *P* value < 0.05 and *q* value < 0.2. A threshold of *P* < 0.05 was used to determine significantly enriched KEGG pathways.

### 2.5. ROC Curve of DIRGs

The receiver operating characteristic (ROC) curves were plotted with the true-positive rate (sensitivity) as the vertical coordinate and the false-positive rate (1−specificity) as the horizontal coordinate. The R package pROC was used to plot the ROC curves for differentially immune-related genes (DIRGs). Its sensitivity and specificity in distinguishing normal samples and MG samples were determined by the AUC value.

### 2.6. Patients and Healthy Controls

Ethical approval for this study was provided by the Research Ethics Committee of the First Affiliated Hospital, Guangxi Medical University. Between July 2021 and October 2021, patients with MG were recruited from the Neurology Clinic of the First Affiliated Hospital, Guangxi Medical University. Patients were confirmed by typical myasthenic symptoms in combination with myasthenia gravis-related antibodies in serum, Tensilon test, or neurophysiological studies according to the China guidelines for the diagnosis and treatment of myasthenia gravis. Patients were eligible if they met the inclusion criteria of 18 to 70 years of age [9]. The exclusion criteria were as follows: (1) patients with severe cardiopulmonary disease; (2) patients with bone marrow function or abnormal coagulation; (3) patients with abnormal liver or kidney function; (4) patients with diabetes or malnutrition; and (5) patients with serious complications, such as infection, malignancies, and other serious organic diseases. Healthy controls were chosen to match the same sex and similar age distributions of MG patients.

### 2.7. Isolation of PBMCs and RNA Extraction

Human peripheral blood mononuclear cells (PBMCs) were isolated from freshly drawn venous blood by density gradient centrifugation over Ficoll-Paque reagent (Solarbio Science & Technology, Beijing, China). According to the manufacturer's procedures, total RNA was extracted using the RNA Extraction Kit (Takara, Dalian, China).

### 2.8. Quantitative Real-Time Polymerase Chain Reaction

CDNA was synthesized by PCR using PrimeScript Kit (TaKaRa, Dalian, China). The primers were designed and synthesized by Sangon Biotech (Shanghai, China). The thermocycling protocol was set according to the manufacturer's instructions, which includes the following steps: initial denaturation for 30 s at 95°C, followed by 40 cycles of denaturation at 95°C for 5 s and 60°C for 34 s. After amplification, a melting curve analysis was performed to evaluate specific amplification. GAPDH mRNA was used as an endogenous control to normalize gene expression levels using the 2-*ΔΔ*CT method. All amplification reactions were performed in triplicate. The primer sequences used for RT-PCR were as follows: DDB1- and CUL4-associated factor 12 (DCAF12), forward primer (GGTATCCATGCCATCGAGCTGAATC) and reverse primer (AACCCCGATGATGGGAAT); heat shock protein family A member 1A (HSPA1A), forward primer (AAGAACGCCCTGGAGTCCTACG) and reverse primer (CCACGAGATGACCTCTTGACACTTG); GAPDH forward primer (GCACCGTCAAGGCTGAGAAC) and reverse primer (TGGTGAAGACGCCAGTGGA).

### 2.9. Statistical Analysis

If the data passed the normality test, the difference in gene mRNA expression between two groups was examined by a parametric test (Student's *t*-test); if not, a Mann–Whitney nonparametric test was used. The diagnostic efficacy of the result gene's mRNA was examined with the use of the ROC curve with *P* < 0.05 defining statistical significance.

## 3. Results

### 3.1. Identification of DEGs

**Figure 1:**
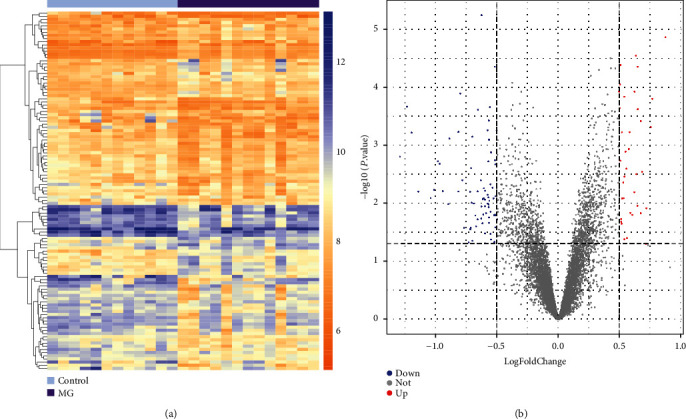
DEG visualization analysis. (a) Heatmap showing the expression levels of the DEGs. The rows represent 25 samples, blue is the healthy control group (*N* = 12), and purple is the MG group (*N* = 13). The vertical axis represents 113 differential genes. The color of the squares represents the expression level of differential genes. (b) Volcano plot of the genes. The *x*- and *y*-axis is log2 fold change and −log10∗adjusted *P* value. Blue and red dots are the downregulated and upregulated genes, respectively. Gray dots are the nondifferentially expressed genes. DEGs are differentially expressed genes.

According to the R limma package and the thresholds of *P* < 0.05 and |logFC| > 0.5, 113 DEGs (76 downregulated and 37 upregulated) were identified in the MG samples compared to the normal samples in the GSE85452 dataset (supplementary table (available here)). As shown in Figure 1, the heatmap (Figure 1(a)) and the volcano plot (Figure 1(b)) showed the expression levels of DEGs in each sample.

### 3.2. Functional Enrichment Analyses of DEGs

**Figure 2:**
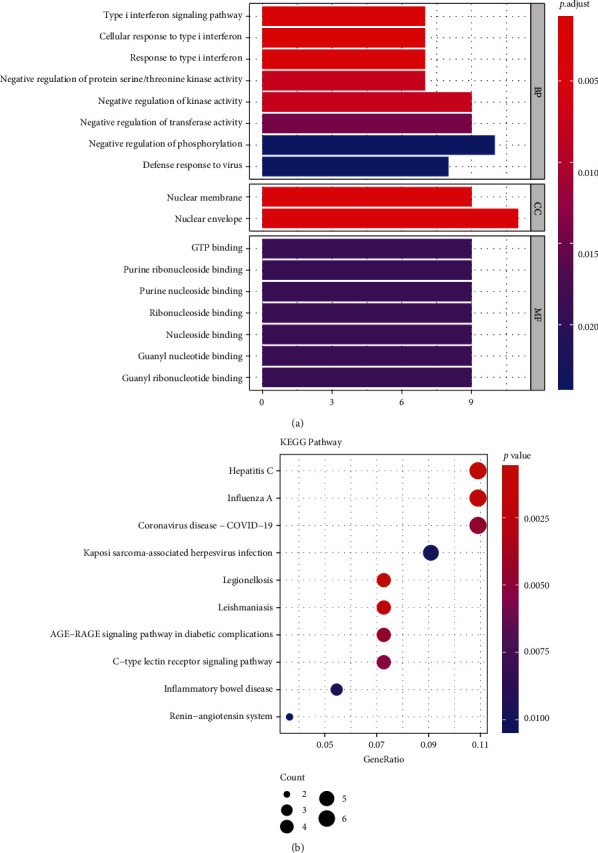
Top 10 enriched GO terms and top 10 KEGG pathways for differentially expressed genes. (a) GO term enrichment analysis. The length of the bar length represents the number of genes; the color of the bar represents *P*. adjust. (b) KEGG pathway analysis. The size of the node size represents the number of genes. Node color represents *P*. adjust. GO is Gene Ontology. BP is the biological process. MF is the molecular function. CC is the cellular component. KEGG is the Kyoto Encyclopedia of Genes and Genomes. *P*. adjust is the adjusted *P* value.

GO and KEGG enrichment analyses were applied to discover the potential biological functions of DEGs. As shown in Figure 2(a), eleven biological processes were enriched, such as the type I interferon signaling pathway, the cellular response to type I interferon, the negative regulation of kinase activity, and the negative regulation of phosphorylation. Two cellular components, the nuclear membrane and the nuclear envelope, were found to be related to DEGs. Seven molecular functions were discovered, involving GTP binding, purine ribonucleoside binding, and ribonucleoside binding. In Figure 2(b), the KEGG pathway results showed that twenty-six pathways related to the disease were enriched, such as hepatitis C, influenza A, legionellosis, and leishmaniasis.

### 3.3. Identification of Immune-Related Hub Genes by WGCNA

**Figure 3:**
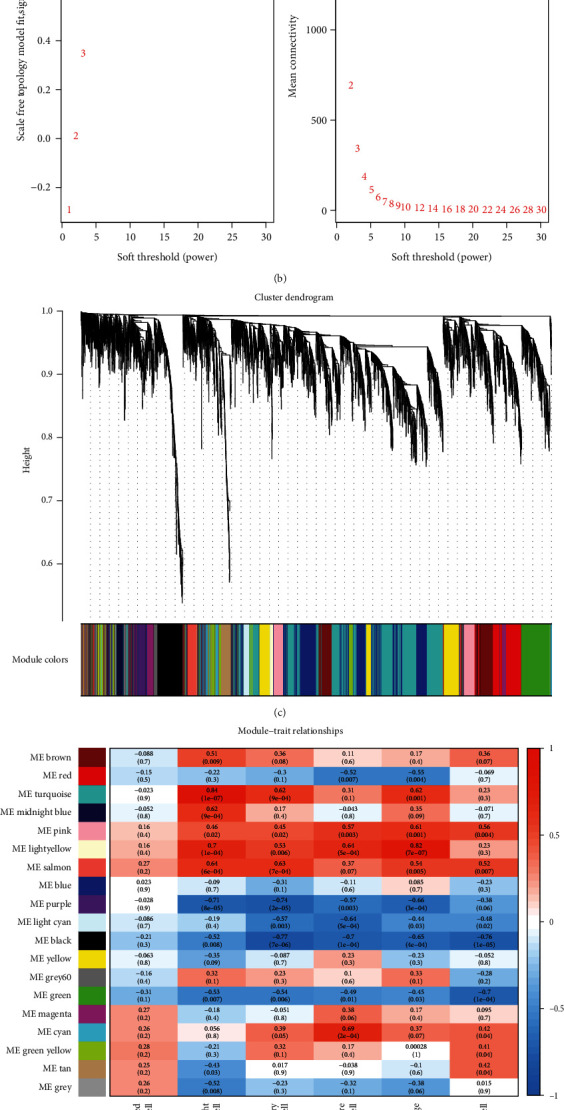
ssGSEA enrichment and WGCNA result overview. (a) Box plots of infiltrating immune cells. The *x*-axis represents the types of immune cells that are significantly and differentially expressed between the normal and MG groups. The *y*-axis represents the ssGSEA enrichment score. Blue and yellow indicate normal samples and MG samples, respectively. (b) Soft threshold screening plot. The soft power of *β* = 7 was selected as the soft threshold for subsequent analyses. (c) Hierarchical clustering tree. The upper part of this figure represents the clustering of genes. The lower part represents the gene modules, which make up 19 modules. Gray represents genes that have not been classified into modules. (d) Heatmap of the correlation between the module eigengenes and clinical traits of MG. Each row represents a different gene module, and each column is a representative trait. The value in the box represents the correlation and the *P* value. Red represents positive correlation, and blue represents negative correlation. (e) Scatter plot of gene significance for CD56^bright^ natural killer cells and MM correlation analysis in the turquoise module.

The R software package GSVA was used to calculate the immune cell enrichment scores of 28 immune cell types. Six types of immune cells with significant differences in enrichment scores between normal and MG samples were then selected as clinical characteristics of WGCNA in the GSE85452 dataset (Figure 3(a)). The six types of immune cells included activated CD8 T cells, CD56^bright^ natural killer cells, immature dendritic cells, effector memory CD8 T cells, macrophages, and mast cells.

The R package WGCNA was used to construct coexpression networks for immune-related hub genes. When the soft threshold *β* was set to 7 (*R*^2^ = 0.85), the gene distribution conformed to the scale-free network (Figure 3(b)). Next, hierarchical clustering divided the coexpression network into 19 modules (Figure 3(c)). The correlation results between 19 modules and clinical characteristics (six immune cells) are shown in Figure 3(d). The correlation between CD56^bright^ natural killer cells and MEturquoise was the most significant, with a correlation coefficient of 0.84 (*P* = 10^−7^). Therefore, the MEturquoise module was selected as the key module for this study. Finally, according to the threshold MM > 0.8 and GS > 0.8, 64 genes highly related to the CD56^bright^ natural killer cells in the MEturquoise module were selected and named immune-related hub genes (Figure 3(e)).

### 3.4. Identification of DIRGs and Functional Enrichment Analyses

**Figure 4:**
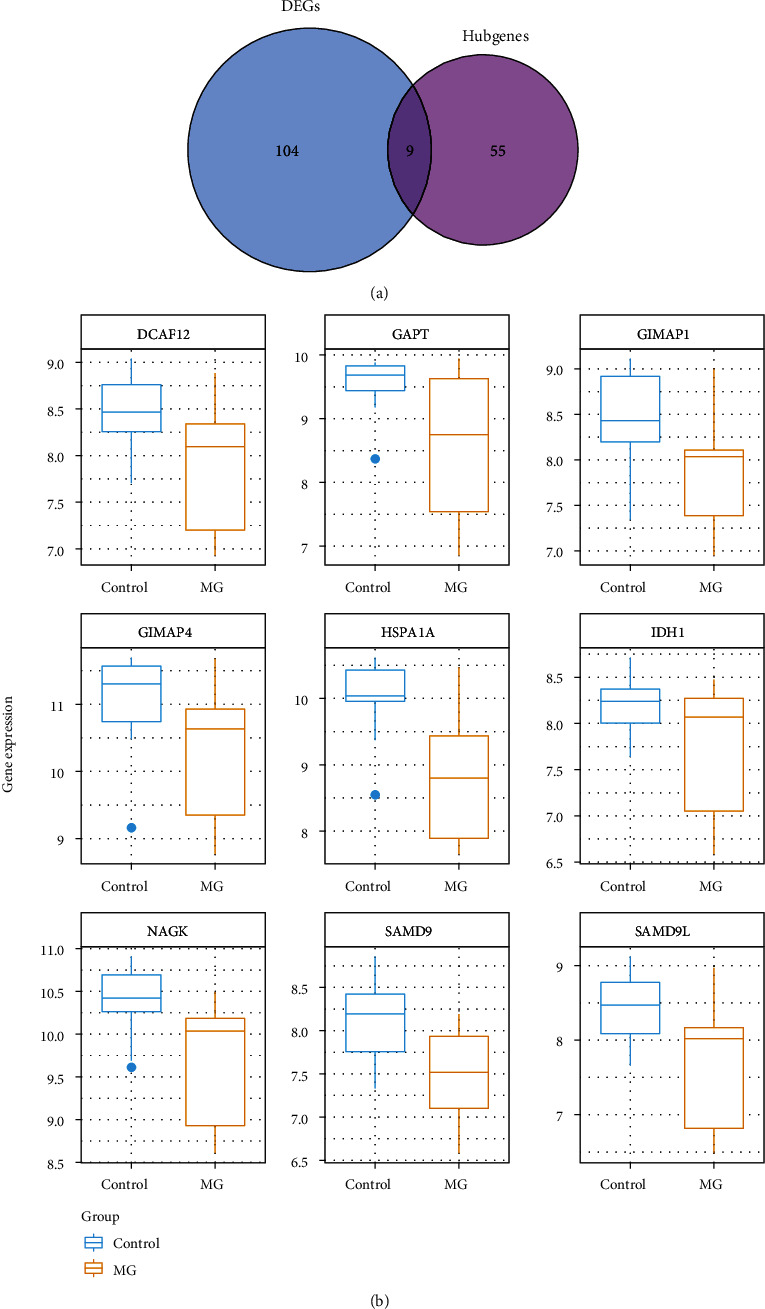
Venn diagram and box plots of DIRGs. (a) Venn diagram of DEGs and hub genes. The blue area represents genes significantly differentially expressed between normal and MG patients (*N* = 113). The purple area represents the hub genes screened by the WGCNA network (*N* = 64). (b) Box plots of DIRGs. The expressions of nine DIRGs were all lower in the MG samples.

There were 9 overlap genes (HSPA1A, SAMD9L, GIMAP1, NAGK, GAPT, IDH1, SAMD9, DCAF12, and GIMAP4) between 113 DEGs and 64 immune-related hub genes (Figure 4(a)). The expressions of 9 DIRGs were all lower in the MG samples (Figure 4(b)).

### 3.5. ROC Curve of DIRGs

**Figure 5:**
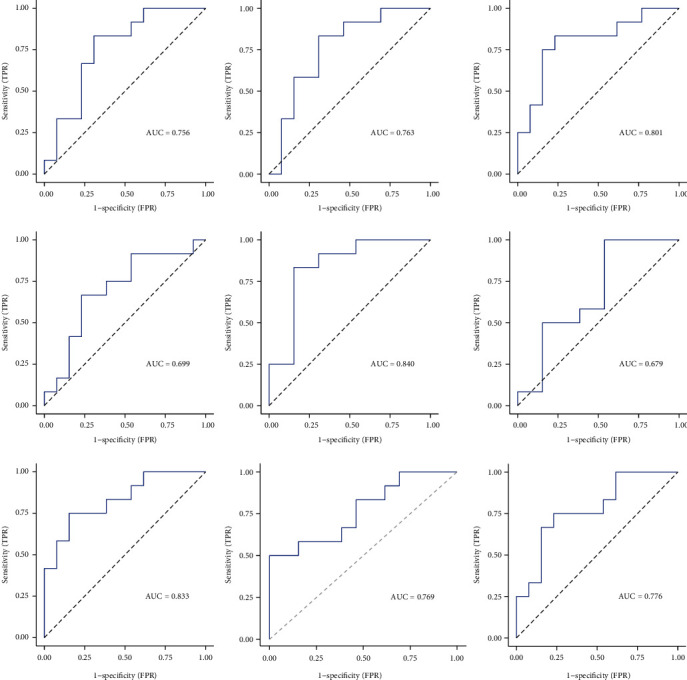
Analysis of the ROC curve of DIRGs. Except for IDH1 and GIMAP4, the AUCs of the other seven genes were greater than 0.7. Among them, heat shock protein family A member 1A (HSPA1A) and N-acetylglucosamine kinase (NAGK) had the most significant AUC values, 0.840 and 0.833, respectively.

ROC curve analysis was performed to evaluate the sensitivity and specificity of the nine DIRGs in distinguishing MG samples. As shown in Figure 5, except for IDH1 and GIMAP4, the AUCs of the other seven genes were greater than 0.7. HSPA1A and NAGK had the most significant AUC values, 0.840 and 0.833, respectively.

### 3.6. Identification of Biomarkers

**Figure 6:**
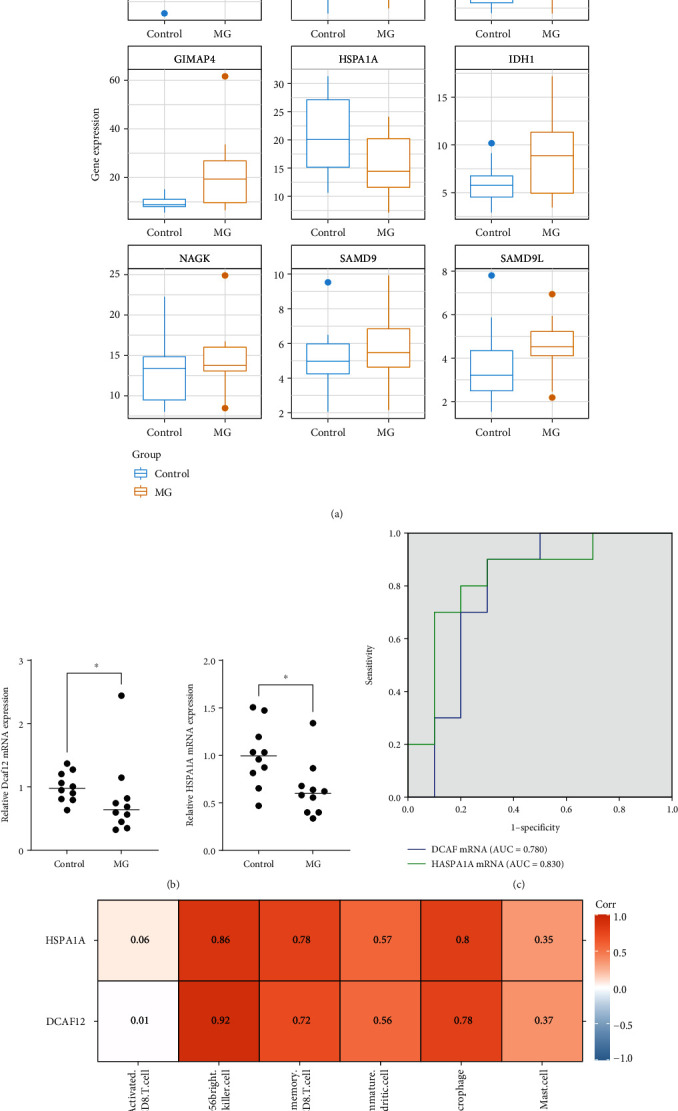
DIRG validation results. (a) Validation results of The Cancer Genome Atlas dataset. The verification dataset results showed that the expression levels of DCAF12 and HSPA1A in MG samples were significantly decreased compared to those in the normal sample, which was consistent with their expression trends in the GSE85452 dataset. (b) The mRNA expression of DCAF12 and HSPA1A. The RNA expression levels of DCAF12 (*P* = 0.035) and HSPA1A (*P* = 0.019) in PBMCs of MG patients were significantly lower than those of healthy controls. (c) ROC curve analysis of DCAF and HSPA1A. (d) Heatmap of the correlation analyses between expression levels (HSPA1A and DCAF12) and six types of immune cells with significant differences quantified by ssGSEA. The expressions of HSPA1A and DCAF12 were all significantly and positively correlated with four immune cell types, namely, the CD56^bright^ natural killer cell, immature dendritic cell, effector memory CD8 T cell, and macrophage.

Using MG and control samples in TCGA-THYM, the expression levels of 9 DIRGs were further verified. The verification dataset results showed that the expression levels of DCAF12 and HSPA1A in MG samples were significantly decreased compared to those in the normal sample, which was consistent with their expression trends in the GSE85452 dataset (Figure 6(a)). Therefore, DCAF12 and HSPA1A were selected as biomarkers for the follow-up study.

To determine the status of DCAF12 and HSPA1A mRNA in PBMCs of MG patients, RNA was extracted from freshly isolated PBMCs of MG patients (*n* = 10) and healthy controls (*n* = 10). The expression levels of the RNA were analyzed by semiquantitative RT-PCR. Baseline characteristics of MG patients and healthy controls are shown in Table 1. The RT-PCR results were consistent with the results of the verification data set, indicating that relative RNA expression levels of DCAF12 (*P* = 0.035) and HSPA1A (*P* = 0.019) RNA expression in PBMC of MG patients were significantly lower than those of healthy controls (Figure 6(b)). The analysis of the ROC curve showed that the ROC curve (AUC) of the expression of DCAF12 and HSPA1A mRNA in the detection of MG was 0.780 (*P* = 0.034) and 0.830 (*P* = 0.013), respectively (Figure 6(c)). The sensitivity, specificity, and Youden index of DCAF12 mRNA expression in the diagnosis of MG were 90.0%, 70.0%, and 0.600, respectively, while those of HSPA1A mRNA expression were 80.0%, 80.0%, and 0.600, respectively. Furthermore, we determined the association between expression levels (DCAF12 and HSPA1A) and six types of immune cells with significant differences quantified by ssGSEA in MG using Spearman correlation (coefficient and *P* value). The results showed that the expressions of DCAF12 and HSPA1A were all significantly and positively correlated with four types of immune cells: the CD56^bright^ natural killer cells (*r* = 0.86 for DACF12, *r* = 0.92 for HSPA1A), the immature dendritic cells (*r* = 0.57 for DACF12, *r* = 0.56 for HSPA1A), the effector memory CD8 T cells (*r* = 0.78 for DACF12, *r* = 0.72 for HSPA1A), and macrophages (*r* = 0.80 for DACF12, *r* = 0.78 for HSPA1A) (*r* > 0.4, *P* < 0.05, Figure 6(d)).

## 4. Discussion

Recently, microarray technology and next-generation sequencing technology have been widely used in medical research, including autoimmune disease research [10, 11]. The innovation of this study is that by combining ssGSEA and WGCNA bioinformatic analysis methods to explore the differences in immune cell types between MG and healthy control from the microarray dataset, we then determine the expression and clinical significance of identified immune-related hub genes the DCAF12 and HSPA1A mRNA in MG. The proportion of CD56^bright^ natural killer (NK) cells in the peripheral blood of MG patients is lower than that of the healthy control. DCAF12 and HSPA1A mRNA are abnormally lowly expressed in MG. The CD56^bright^ NK cells and immune-related hub genes (DCAF12 and HSPA1A) were the most correlated. DCAF12 and HSPA1A may be critical in the occurrence of MG by affecting the number and function of the CD56^bright^ natural killer (NK) cell. In addition, DCAF12 and HSPA1A can be used as candidate markers for the diagnosis of MG. Lower expression of DCAF12 and HSPA1A indicates an increased risk of MG occurrence.

Although current research on autoimmune diseases has mainly focused on T and B lymphocytes, it has been recognized that NK cells participate in and maintain an adaptive immune response or peripheral tolerance mechanism. Natural killer cells account for about 5-15% of lymphocytes in human peripheral blood. According to the expression level of CD56, NK cells can be divided into CD56^bright^ NK cells and CD56^dim^ NK cells. CD56^bright^ NK cells account for about 10% of NK cells in peripheral blood and express CD56 highly. CD56^bright^ NK cells exert immune regulation by secreting interferon-*γ*, tumor necrosis factor-*α*, and interleukin-10. They have the function of avoiding excessive inflammation or the development of autoimmune reactions. Up to now, NK cell studies in MG are rare. A recent study has found an abnormal phenotype and function of NK cells in patients with MG [12]. Consistent with previous studies, our findings have found that, compared with healthy controls, the proportion of CD56^bright^ NK cells in the peripheral blood of MG patients is lower [12]. The role of CD56^bright^ NK cells is to prevent the overactivation of CD4+ T cells through cytotoxicity [13]. In MG patients, the killing effect of NK cells against CD4+ T cells and T follicular helper cells (Tfh) was attenuated, whereas the differentiation and activation of Tfh by NK cells were enhanced [12]. The immune regulation of NK cells to CD4+ T cells and T follicular helper cells is abnormal in MG, which may be one of the immunopathological mechanisms of MG [12]. The detailed immune mechanism of NK cells in MG still requires further research.

Thus far, there is no research on the HSPA1A gene of PBMCs in MG. The HSPA1A gene encodes heat shock 70 kDa protein 1 (HSP70-1). As a member of the HSP70 superfamily, HSP70-1 functions primarily as a molecular chaperone that prevents misfolded protein aggregation. It plays an essential role in maintaining cellular homeostasis in response to cellular stress conditions such as inflammation, oxidative stress, hypo- or hyperoxia, hypo- or hyperthermia, and infections. HSP70-1 has the function of modulating inflammation and antiapoptosis [14, 15]. HSP70 plays an opposite role in the intracellular and extracellular immune response through the NF-*κ*B pathway [16]. The NF-*κ*B pathway plays a critical role in modulating the inflammatory response, including in autoimmune diseases such as MG, whose activity is dysregulated [17]. Endogenous HSP70 is involved in the anti-inflammatory process [18]. A large amount of data had demonstrated that intracellular Hsp70 downregulates NF-*κ*B activity by inhibiting phosphorylation [16, 19]. Furthermore, previous studies have shown that increasing endogenous HSP70 can regulate the number and function of T-reg and B-reg. It can also alleviate autoimmune diseases [20, 21]. A study of Behçet's syndrome found that the HSPA1A protein level of active patients' PBMCs was significantly lower than that of healthy controls [22]. Our RT-PCR results and previous research findings suggest that the decreased level of HSPA1A transcription expression of PBMCs cells may be part of the immunological pathogenesis of MG. The underlying mechanism may be that the decreased endogenous expression of HSPA1A causes the protein folding disorder and increases the aggregation of misfolded or unfolded proteins in PBMCs, then leads to an impairment of the number and function of PBMC cell subsets, which in turn triggers the occurrence of MG.

DCAF12 is a substrate recognition receptor of the CUL4-DDB1 E3 ubiquitin-protein ligase complex and is involved in various cellular processes, including cell cycle progression, signal transduction, apoptosis, and gene regulation [23]. Furthermore, DCAF12 is related to several immune processes. Transcriptome analysis of blood before and after hepatitis B vaccination showed that the level of DCAF12 in nonresponders was significantly upregulated after immunization [24]. In previous clinical studies, the decrease in DCAF12, also called WDR40A, in peripheral blood preceded organ rejection after organ transplantation [25, 26]. DCAF12 is related to the development of intestinal Behçet's disease and chronic inflammatory diseases [27]. Various immune cell populations in the spleen of DCAF12 knockout mice are dysregulated, among which T cell populations are the most dysregulated. Furthermore, the lack of DCAF12 will also affect the physiological state of activated T cells [27]. The bioinformatic analysis results of this study showed that the DCAF12 expression level of PBMCs was significantly lower than that of healthy controls and was confirmed by the RT-PCR experiment. Our findings indicate that the decline of the DACF12 transcription level may be one of the immunological mechanisms of the MG. The decrease in the expression level of DCAF12 leads to an enhanced inflammatory response, which may easily induce the occurrence of MG. Interestingly, studies have shown that presynaptic DCAF12 is necessary to induce neurotransmitter release and homeostatic synaptic enhancement in the larval neuromuscular junction. At the same time, postsynaptic DCAF12 maintains postsynapse glutamate receptor IIA, IIC, and IID levels [28]. Studying the expression levels of presynaptic and postsynaptic DCAF12 in the MG neuromuscular junction may be a potential future research direction, which will help understand the relationship between the level of DCAF12 in the neuromuscular junction and MG.

At present, the clinical diagnosis of MG has based chiefly on the examination results of circulating autoantibody detection of NMJ antigens. However, some patients with MG are seronegative with these classical commercial serologic tests. Moreover, the efficiency of electrophysiological tests used in recent years is not very high, which often causes mildly painful feeling at the time of the examination and postelectrophysiology procedure pain after that [29]. Bedside tests such as fatigue test, eyes-closed resting test, and orbital cooling (ice test) are also not ideal choices for diagnosing MG, with a high false-positive rate [30]. These deficiencies may lead to a higher rate of missed diagnosis, thus affecting the treatment and prognosis. In this study, the AUC of PBMC DCAF12 and HSPA1A mRNA expression in MG diagnosis was 0.780 and 0.830, suggesting a good diagnostic effect. Our results suggest that detecting the levels of DCAF12 and HSPA1A mRNA levels of PBMCs expressed in suspected patients with MG in clinical practice can diagnose MG early and timely treatment measures can be performed. Immune-related hub genes in MG were identified and verified to provide new diagnostic markers for myasthenia gravis, which is essential for subsequent clinical treatment and improving the prognosis of the disease.

Due to the small number of available datasets and insufficient patient samples, subgroup analysis could not be performed, and hub genes were not fully identified, resulting in omissions. We only investigated the transcription level of hub genes and failed to ascertain the protein expression level and biological functions. Finally, the diagnostic efficacy of HSPA1A and DCAF12 mRNA was not compared with common diagnostic indicators for MG. In future studies, we will continue collecting PBMCs, serum samples, and clinical information from MG patients; conducting subgroup analysis; assessing the protein expression levels; exploring biological mechanisms of immune-related hub genes; and comparing with other conventional diagnostic indicators.

In conclusion, we show that RNA expression levels of HSPA1A and DCAF12 in the PBMC of MG patients are lower than those of healthy controls. The proportion of CD56^bright^ NK cells in the peripheral blood from MG patients is low. Low expression of DCAF12 and HSPA1A may increase MG development risk by affecting the number and function of CD56^bright^ natural killer cells. They can distinguish MG patients from healthy controls with high accuracy.

## Figures and Tables

**Table 1:** Baseline characteristics (mean ± SD or %) among MG patients and their matched controls.

	Patients (*n* = 10)	Control (*n* = 10)	*P* value
Sex			
Males	3	3	
Females	7	7	
Age (years), mean ± SD	37.8 ± 12.3	37.4 ± 11.3	0.94
Anti-AChR-Ab-positive	9 (90%)	0	
Anti-MUSK-Ab-positive	1 (10%)	0	
Thymoma	2 (20%)	0	
Symptom duration (months), mean ± SD	63.8 ± 11.2	—	

AChR: acetylcholine receptor; MuSK: muscle-specific tyrosine kinase.

## Data Availability

Data is available at https://www.4shared.com/s/fN_PRsKhoea.

## Supplementary Materials

Supplementary Materials Supplementary Table 1: the differentially expressed genes (DEGs) were identified in the MG samples compared to the normal samples in the GSE85452 dataset. This supplementary table contains 113 DEGs, of which 76 were downregulated and 37 were upregulated.
