# Supplementary material for: DCAF12 and HSPA1A May Serve as Potential Diagnostic Biomarkers for Myasthenia Gravis
Source: Biomed Res Int. 2022 May 24;2022:8587273. doi: 10.1155/2022/8587273 (PMC9155969; doi:10.1155/2022/8587273)
Supplement: Supplementary Materials — Supplementary Table 1: the differentially expressed genes (DEGs) were identified in the MG samples compared to the normal samples in the GSE85452 dataset. This supplementary table contains 113 DEGs, of which 76 were downregulated and 37 were upregulated. [file 8587273.f1.doc]

|  | logFC | AveExpr | t | P.Value | adj.P.Val | B | change |
| --- | --- | --- | --- | --- | --- | --- | --- |
| ERICH1 | -0.62493215 | 9.158593472 | -5.6340105 | 5.76E-06 | 0.041608134 | 3.827652031 | DOWN |
| ZNF296 | 0.876752212 | 8.603853094 | 5.304720729 | 1.38E-05 | 0.041608134 | 3.066210934 | UP |
| CLP1 | 0.636275369 | 6.989242972 | 5.032995524 | 2.86E-05 | 0.041608134 | 2.431995557 | UP |
| MAD2L1BP | 0.510603695 | 7.093356877 | 4.893748658 | 4.14E-05 | 0.041608134 | 2.105585137 | UP |
| CHIC2 | -0.517090403 | 8.77809937 | -4.867813932 | 4.44E-05 | 0.041608134 | 2.044718752 | DOWN |
| YOD1 | 0.651424929 | 7.475865097 | 4.86729763 | 4.45E-05 | 0.041608134 | 2.043506849 | UP |
| SLC30A1 | 0.507500118 | 7.777588648 | 4.60643945 | 8.94E-05 | 0.057074042 | 1.430645187 | UP |
| DUSP12 | 0.626275158 | 8.33281884 | 4.501559251 | 0.000118328 | 0.061229591 | 1.184219126 | UP |
| KTI12 | 0.501290111 | 7.737888684 | 4.50100737 | 0.000118503 | 0.061229591 | 1.182922866 | UP |
| MX2 | -0.79677413 | 8.935810506 | -4.469427955 | 0.000128929 | 0.061229591 | 1.108759854 | DOWN |
| CDKN2D | 0.543914567 | 7.804636852 | 4.422649978 | 0.000146071 | 0.063442744 | 0.998949606 | UP |
| ING1 | 0.769421362 | 8.040024081 | 4.391220606 | 0.000158842 | 0.063442744 | 0.925207178 | UP |
| SERPINB2 | -1.229184763 | 7.435457433 | -4.277056719 | 0.000215276 | 0.063442744 | 0.65767691 | DOWN |
| CEMIP2 | -0.556593641 | 7.741044653 | -4.268637353 | 0.00022015 | 0.063442744 | 0.637971671 | DOWN |
| FEM1B | 0.50416938 | 6.934438086 | 4.254424781 | 0.000228628 | 0.063442744 | 0.604716439 | UP |
| HHEX | 0.651856737 | 8.834139997 | 4.238009681 | 0.000238822 | 0.063442744 | 0.566321909 | UP |
| IRS2 | -0.653499225 | 9.311621028 | -4.231225018 | 0.000243165 | 0.063442744 | 0.550457369 | DOWN |
| NCOA7 | -0.565641067 | 8.635460895 | -4.067702777 | 0.000375056 | 0.072880578 | 0.169054186 | DOWN |
| KLF11 | 0.675940973 | 8.768334709 | 4.062243474 | 0.000380506 | 0.072880578 | 0.156357083 | UP |
| PPP1R2 | 0.757042137 | 8.99645062 | 3.969604743 | 0.000485832 | 0.078293773 | -0.05867384 | UP |
| YWHAG | -0.574452655 | 8.127818529 | -3.920140082 | 0.000553336 | 0.079295463 | -0.173129921 | DOWN |
| DOCK10 | -0.501562187 | 8.97076449 | -3.919560276 | 0.00055418 | 0.079295463 | -0.17446992 | DOWN |
| C3AR1 | -0.811614837 | 7.596998836 | -3.897366374 | 0.000587438 | 0.080853147 | -0.225733402 | DOWN |
| ZDHHC1 | 0.585950159 | 8.213490028 | 3.893017145 | 0.000594181 | 0.080853147 | -0.235772486 | UP |
| SMDT1 | 0.52320618 | 7.188794703 | 3.89294521 | 0.000594294 | 0.080853147 | -0.235938511 | UP |
| HSPA1A | -1.192351342 | 9.390419062 | -3.886453232 | 0.000604503 | 0.080915662 | -0.250919331 | DOWN |
| FPR2 | -0.699070614 | 7.443016983 | -3.826268301 | 0.000707733 | 0.086716824 | -0.389553107 | DOWN |
| EGR2 | -0.885751132 | 9.059839799 | -3.794662855 | 0.000768678 | 0.088304862 | -0.462166501 | DOWN |
| NABP1 | -0.563727243 | 7.953655571 | -3.780307175 | 0.000798032 | 0.088304862 | -0.495103478 | DOWN |
| PIK3IP1 | 0.579269744 | 8.013748452 | 3.637059704 | 0.001157996 | 0.111503067 | -0.822077763 | UP |
| SERTAD2 | 0.552019987 | 9.372038075 | 3.596037576 | 0.001287511 | 0.114893069 | -0.915098998 | UP |
| LFNG | -0.547549846 | 9.317907301 | -3.585944042 | 0.001321482 | 0.115441889 | -0.937941513 | DOWN |
| PTGS2 | -1.28684656 | 9.657985797 | -3.520380724 | 0.001564367 | 0.124833452 | -1.085859368 | DOWN |
| MYOF | -0.515400741 | 7.834395189 | -3.462575065 | 0.001814098 | 0.130374313 | -1.215583223 | DOWN |
| GID8 | 0.509026765 | 8.940117135 | 3.457113452 | 0.001839602 | 0.130374313 | -1.227804687 | UP |
| DYNLL1 | -0.977901692 | 9.620959557 | -3.447930768 | 0.001883267 | 0.130374313 | -1.248338766 | DOWN |
| PREP | -0.569691942 | 7.436067566 | -3.447537894 | 0.001885157 | 0.130374313 | -1.249216904 | DOWN |
| IL1B | -0.963699874 | 10.91682753 | -3.40866201 | 0.0020816 | 0.135027787 | -1.335948098 | DOWN |
| RNASEL | -0.513129199 | 7.184062113 | -3.402380412 | 0.002115148 | 0.135027787 | -1.349931431 | DOWN |
| NAGK | -0.708263151 | 10.03105531 | -3.342601546 | 0.002461723 | 0.143526256 | -1.482559909 | DOWN |
| CIRBP | 0.559702061 | 9.739987794 | 3.333099544 | 0.002521641 | 0.145327064 | -1.503565625 | UP |
| RUNX3 | 0.686771879 | 9.193053198 | 3.281503238 | 0.002872388 | 0.153793238 | -1.617249365 | UP |
| SNHG9 | 0.640306354 | 8.259981772 | 3.247181682 | 0.003131269 | 0.157493358 | -1.692506278 | UP |
| C16orf87 | 0.544181749 | 7.755888424 | 3.208838687 | 0.003447037 | 0.163468329 | -1.776222598 | UP |
| EGR3 | -0.779585764 | 6.999203871 | -3.149824455 | 0.003993563 | 0.170136001 | -1.904302111 | DOWN |
| FRAT2 | -0.630050936 | 9.501618064 | -3.149412769 | 0.003997653 | 0.170136001 | -1.905192227 | DOWN |
| NUAK2 | 0.534212447 | 7.397760606 | 3.113067182 | 0.004374978 | 0.173750567 | -1.983586369 | UP |
| CHMP1B | -0.552518151 | 10.43567415 | -3.085732311 | 0.004680976 | 0.179085177 | -2.042293766 | DOWN |
| LEPROTL1 | -0.536044668 | 8.088815684 | -3.031533347 | 0.005349244 | 0.186914207 | -2.158037426 | DOWN |
| CEPT1 | -0.606327116 | 7.889180972 | -3.018624185 | 0.005521338 | 0.186914207 | -2.18547253 | DOWN |
| GIMAP8 | -1.010600316 | 8.367761297 | -2.990548135 | 0.005914017 | 0.186914207 | -2.244959867 | DOWN |
| SIK1 | -0.595270199 | 7.948831394 | -2.974382146 | 0.006152031 | 0.186914207 | -2.279097953 | DOWN |
| PDE4B | -0.937956936 | 7.796269993 | -2.970528834 | 0.006210094 | 0.186914207 | -2.287222592 | DOWN |
| TAOK1 | -0.65632588 | 7.165809989 | -2.966126441 | 0.006277068 | 0.186914207 | -2.296499039 | DOWN |
| CX3CR1 | -0.81052514 | 9.303388847 | -2.962608273 | 0.006331083 | 0.186914207 | -2.303907747 | DOWN |
| CYBB | -1.137124612 | 9.676435208 | -2.959552656 | 0.006378355 | 0.186914207 | -2.310339103 | DOWN |
| EPSTI1 | -0.71757634 | 8.97308784 | -2.957387324 | 0.006412056 | 0.186914207 | -2.314894761 | DOWN |
| CLEC2B | 0.624108417 | 7.441592855 | 2.956613208 | 0.006424146 | 0.186914207 | -2.316523055 | UP |
| CAPZA1 | -0.574503971 | 7.803847232 | -2.909798905 | 0.007196854 | 0.193319539 | -2.414622006 | DOWN |
| COPB2 | -0.5122912 | 8.478334823 | -2.887811991 | 0.007589447 | 0.193767479 | -2.460438637 | DOWN |
| TBC1D9 | -0.568151155 | 8.160590488 | -2.86546967 | 0.008009102 | 0.195418281 | -2.506823821 | DOWN |
| ANKDD1A | 0.535839522 | 6.778143532 | 2.857637136 | 0.008161346 | 0.195825795 | -2.523043447 | UP |
| CXCL2 | -1.037724395 | 7.685128032 | -2.85563232 | 0.008200752 | 0.195825795 | -2.527191524 | DOWN |
| RNF10 | 0.524712714 | 7.886063633 | 2.853115048 | 0.008250485 | 0.195825795 | -2.532397876 | UP |
| SAMD9 | -0.527701755 | 7.816287162 | -2.851718073 | 0.008278208 | 0.195825795 | -2.535286197 | DOWN |
| ISG15 | -0.608453684 | 8.300562508 | -2.851511965 | 0.008282306 | 0.195825795 | -2.535712278 | DOWN |
| TLR5 | -0.614135999 | 7.933825638 | -2.838151265 | 0.008552054 | 0.198805307 | -2.56329996 | DOWN |
| ANPEP | -0.596867643 | 8.092252236 | -2.829179482 | 0.008737824 | 0.201194227 | -2.581789157 | DOWN |
| PCMT1 | -0.560751655 | 9.627649767 | -2.809751841 | 0.009153224 | 0.203198682 | -2.62172557 | DOWN |
| SAMD9L | -0.704514774 | 8.056868316 | -2.779093444 | 0.009846821 | 0.203321366 | -2.684465029 | DOWN |
| MX1 | -0.68630403 | 10.41868457 | -2.765206292 | 0.010177013 | 0.203321366 | -2.712767785 | DOWN |
| RHOB | -0.573667456 | 10.16466847 | -2.762729402 | 0.010236988 | 0.203321366 | -2.717808137 | DOWN |
| GAPT | -0.89386997 | 9.071037432 | -2.75442689 | 0.010440459 | 0.203321366 | -2.734686278 | DOWN |
| DCAF12 | -0.567131261 | 8.174549632 | -2.74755891 | 0.01061164 | 0.203321366 | -2.748628213 | DOWN |
| CXXC5 | 0.649202429 | 8.762955885 | 2.732696065 | 0.010991153 | 0.203321366 | -2.778737365 | UP |
| RGS18 | -0.596726573 | 9.403987739 | -2.719839892 | 0.011329663 | 0.203620179 | -2.804712118 | DOWN |
| DPYD | -0.62442044 | 9.213186593 | -2.716546365 | 0.011417944 | 0.203620179 | -2.811355981 | DOWN |
| GIMAP1 | -0.567005598 | 8.174588862 | -2.703856202 | 0.011764159 | 0.204676634 | -2.836915205 | DOWN |
| E2F2 | 0.722653918 | 8.020328046 | 2.69013546 | 0.012149541 | 0.207894928 | -2.864478185 | UP |
| IFI44L | -0.551754316 | 7.419953762 | -2.628733191 | 0.014023307 | 0.215009566 | -2.986892256 | DOWN |
| CARD19 | 0.591512579 | 8.226955984 | 2.612602716 | 0.014558469 | 0.218482338 | -3.018792091 | UP |
| XYLT1 | 0.675539014 | 7.043041182 | 2.606270679 | 0.014773699 | 0.219062341 | -3.031284454 | UP |
| LPXN | -0.587003656 | 9.78531363 | -2.602665161 | 0.014897573 | 0.219209972 | -3.038390127 | DOWN |
| RAB10 | -0.610366339 | 10.211349 | -2.578926926 | 0.015737571 | 0.22322881 | -3.085034396 | DOWN |
| GCA | -0.514886686 | 10.13305293 | -2.577554563 | 0.015787454 | 0.22322881 | -3.087723611 | DOWN |
| ID1 | 0.606464945 | 7.79989252 | 2.577396192 | 0.01579322 | 0.22322881 | -3.088033895 | UP |
| TMEM176B | -0.521695161 | 6.760276387 | -2.568551301 | 0.016118369 | 0.223840381 | -3.105345756 | DOWN |
| MNDA | -0.673938676 | 10.39292289 | -2.563557554 | 0.016304679 | 0.224782369 | -3.11510488 | DOWN |
| FAR1 | -0.509520924 | 8.140448931 | -2.563250546 | 0.016316198 | 0.224782369 | -3.115704502 | DOWN |
| STK38 | -0.563982952 | 9.273188222 | -2.527224918 | 0.017721531 | 0.228737569 | -3.185779222 | DOWN |
| DUSP2 | -0.6263846 | 8.666394994 | -2.511588077 | 0.018365719 | 0.233768558 | -3.216015597 | DOWN |
| ELF4 | 0.517128147 | 8.914990843 | 2.506889675 | 0.018563468 | 0.234951435 | -3.225079253 | UP |
| ACTR3 | -0.65579812 | 9.452397901 | -2.496270662 | 0.019017677 | 0.236435629 | -3.245527528 | DOWN |
| IDH1 | -0.500105443 | 7.929760504 | -2.489964098 | 0.019292265 | 0.237194823 | -3.257647375 | DOWN |
| GPR1 | 0.517841781 | 7.507458872 | 2.454979794 | 0.020883054 | 0.242035464 | -3.324547892 | UP |
| HCP5 | -0.565195218 | 9.306342492 | -2.434278399 | 0.021880385 | 0.244033269 | -3.363867072 | DOWN |
| RTN1 | -0.616398859 | 9.168155709 | -2.426251681 | 0.022278732 | 0.244033269 | -3.379058286 | DOWN |
| MIR22HG | 0.521012991 | 9.208660131 | 2.422969221 | 0.022443543 | 0.244033269 | -3.385261804 | UP |
| CD52 | -0.668784836 | 9.710859395 | -2.373273459 | 0.025079314 | 0.251502651 | -3.478549825 | DOWN |
| XPO1 | -0.51275459 | 8.908241027 | -2.355831238 | 0.026069605 | 0.255306828 | -3.511007037 | DOWN |
| IFIT1 | -0.711948148 | 7.697091382 | -2.344638228 | 0.02672377 | 0.257066456 | -3.531756248 | DOWN |
| GIMAP4 | -0.76620724 | 10.64677364 | -2.344482741 | 0.026732962 | 0.257066456 | -3.532044046 | DOWN |
| NR4A2 | -0.71586611 | 9.187948195 | -2.324263151 | 0.027953133 | 0.25833302 | -3.56936638 | DOWN |
| GIMAP7 | -0.597031614 | 7.630602972 | -2.245720112 | 0.033188485 | 0.272974464 | -3.712368709 | DOWN |
| CCR1 | -0.552727794 | 8.557143204 | -2.23827395 | 0.033728241 | 0.274961365 | -3.725759439 | DOWN |
| DUSP19 | 0.563220858 | 8.31405255 | 2.166465877 | 0.039354478 | 0.289285046 | -3.853372421 | UP |
| IER5 | 0.543141228 | 9.327493587 | 2.144181601 | 0.041264331 | 0.293983146 | -3.892403535 | UP |
| RPPH1 | -0.547305493 | 7.713022363 | -2.133519369 | 0.042207135 | 0.296342649 | -3.910981068 | DOWN |
| EGR1 | -0.697259762 | 11.24908715 | -2.11003288 | 0.044352124 | 0.301295591 | -3.951678457 | DOWN |
| IFIT2 | -0.520341021 | 8.509606972 | -2.086946926 | 0.046554705 | 0.305840711 | -3.99137758 | DOWN |
| HLA-DRB4 | -0.727948216 | 9.290221173 | -2.081404436 | 0.047097774 | 0.305840711 | -4.000863274 | DOWN |
| MAT2A | -0.595815883 | 9.390616074 | -2.060316955 | 0.049215779 | 0.309055441 | -4.036791786 | DOWN |
| PIM1 | 0.716950022 | 8.204354056 | 2.05666203 | 0.049591344 | 0.310142097 | -4.042992824 | UP |
